# The Association of Metabolic Dysfunction and Mood Across Lifespan Interacts With the Default Mode Network Functional Connectivity

**DOI:** 10.3389/fnagi.2021.618623

**Published:** 2021-08-02

**Authors:** Carlos Portugal-Nunes, Joana Reis, Ana Coelho, Pedro Silva Moreira, Teresa Costa Castanho, Ricardo Magalhães, Paulo Marques, José Miguel Soares, Liliana Amorim, Pedro Guimarães Cunha, Nadine Correia Santos, Patrício Costa, Joana Almeida Palha, Nuno Sousa, João Miguel Bessa

**Affiliations:** ^1^Life and Health Sciences Research Institute (ICVS), School of Health Sciences, University of Minho, Braga, Portugal; ^2^ICVS/3B's, PT Government Associate Laboratory, Braga/Guimarães, Portugal; ^3^Clinical Academic Center—Braga, Braga, Portugal; ^4^Psychological Neuroscience Lab, CIPsi, School of Psychology, University of Minho, Braga, Portugal; ^5^Centro Hospitalar do Alto Ave—EPE, Guimarães, Portugal

**Keywords:** metabolic dysfunction, mood, age, functional connectivity, default mode network

## Abstract

**Background:** Numerous studies suggest a relationship between depression and metabolic syndrome, which is likely influenced by age. Interestingly, functional imaging analysis has shown an association between functional connectivity in the default mode network (DMN-FC) and components of metabolic syndrome, which is explored in this study.

**Methods:** From a larger longitudinal cohort study on healthy aging, 943 individuals were extensively characterized for mood and cognition. Among these, 120 individuals who were selected for displaying extreme cognitive performance within the normal range (good and poor performers) were further studied. Here, in a cross-sectional design, using confirmatory factor analysis (CFA), the association between metabolic dysfunction and depressive mood as a function of age and its relationship with DMN-FC was studied.

**Results:** Metabolic dysfunction was modeled as a second-order latent variable using CFA. First-order latent variables were obesity, glucose dysmetabolism, lipids imbalance, and blood pressure. Using multiple linear regression models, this study observed that metabolic dysfunction, glucose dysmetabolism, and lipids imbalance were linearly associated with depressive mood, and the association with obesity was U-shaped. The association of metabolic dysfunction, obesity, and glucose dysmetabolism with depressive mood is positive for the younger individuals in our sample and vanishes with aging. The FC of the right superior temporal gyrus with the DMN correlated with both obesity and depressive mood. In participants with higher obesity scores, FC increased with higher GDS scores, while in those with lower GDS scores, FC decreased. Age and blood pressure were associated with a more complex pattern of association between FC of the right supramarginal gyrus and GDS score.

**Conclusion:** The association of metabolic dysfunction with depressive mood is influenced by age and relates with differential patterns of DMN-FC. The combination of the effects of age, mood, and metabolic dysfunction is likely to explain the heterogeneity of DMN-FC, which deserves further investigation with larger and longitudinal studies.

## Introduction

Depression is a highly prevalent mood disorder, affecting an estimated 300 million people worldwide (Patel et al., 2016; Herrman et al., 2018). It is projected to become the leading cause of burden of disease by 2030 (Mathers et al., 2008). Symptoms of depression can be present even in the absence of formal criteria to diagnose major depression. Depressive symptoms are widespread in the elderly. Their impact on cognitive and physical decline are similar to those resulting from a variety of other medical and psychiatric conditions (Meeks et al., 2011).

Metabolic syndrome (MetS) is a cluster of metabolic abnormalities associated with high risk of developing type 2 diabetes and/or cardiovascular disease. According to the International Diabetes Federation, it is defined by the presence of visceral adiposity and at least two of the following conditions: hyperglycemia, dyslipidemia (high triglycerides and/or low HDL cholesterol), and hypertension (Alberti et al., 2005). Aging is associated with an increased deposition of body fat in the abdominal region (St-Onge and Gallagher, 2010) and several components of the MetS are more prevalent in older than younger adults.

Several cross-sectional studies have associated depression or depressive symptoms with various components of MetS. Specifically, depression and/or depressive mood have been associated with obesity (de Wit et al., 2010; Luppino et al., 2010), poor glycemic control (Lustman et al., 2000), insulin resistance, (Pearson et al., 2010) and blood pressure has been associated with depressive mood (Paterniti et al., 2000; Lenoir et al., 2008). Data are inconsistent on the relation of dyslipidemia components with mood (Huang, 2005; van Reedt Dortland et al., 2010; Vargas et al., 2014; Beydoun et al., 2015).

Of notice, the association between MetS and depression seems to be bidirectional. Pan et al. (2012), in a meta-analysis that included 155,333 subjects, demonstrated that MetS was associated with depression, that baseline MetS could predict the risk of developing depression, and that the reverse is also true. Analyses were influenced by MetS definitions [National Cholesterol Education Programs Adult Treatment Panel III (NCEP ATP-III) *versus* International Diabetes Federation (IDF)] and depression measures (diagnostic interview *versus* self-reported symptom scale).

MetS constitute a heterogeneous metabolic group and the contribution of each of its components differs between individuals. CFA has been used to construct a hierarchical four-factor model that represents MetS by insulin resistance, obesity, lipids, and blood pressure, which may help to determine the contribution of each component to the overall syndrome (Shen et al., 2003, 2006; Levin et al., 2014).

Since MetS and its components are measures of peripheral metabolic dysfunction and depression is an abnormality of the central nervous system, it is important to explore the impact of peripheral metabolic alterations on brain function and connectivity. The DMN has been well-studied, both in general and in the context of depressive symptomatology. There have been several reports of higher functional connectivity (FC) within the DMN, as well as between the DMN and other brain regions in patients with depression (Whitfield-Gabrieli and Ford, 2012; Kaiser et al., 2015). In contrast, age-associated reduction in DMN-FC has been frequently reported in the population over 60 years of age (Damoiseaux et al., 2008; Soares et al., 2017). Several lines of evidence suggest that individuals with metabolic disorders display alterations in DMN activity and FC (Cha et al., 2014) and that multiple factors, such as age, mood, and metabolic abnormality may interact with one another to produce alterations on DMN-FC.

The present study proposes to (1) utilize the latent variable model of MetS, referred to as metabolic dysfunction; (2) explore, in a cross-sectional investigation, the potential association of metabolic dysfunction and its components with depressive mood in older individuals; (3) assess the impact of advancing age in the strength of those associations; and (4) evaluate the impact of the interaction between metabolic dysfunction, age and mood upon DMN-FC.

## Materials and Methods

### Ethics Statement

The study was conducted in accordance with the Declaration of Helsinki (59th Amendment) and was approved by the Portuguese national ethical committee (Comissão Nacional de Proteção de Dados) and by the local ethics review boards (Hospital de Braga, Braga; Centro Hospitalar do Alto Ave, Guimarães). The study goals and the psychological and clinical assessments were explained to the participants, of whom all gave informed consent.

### Characterization of the Cohort

**Figure 1:**
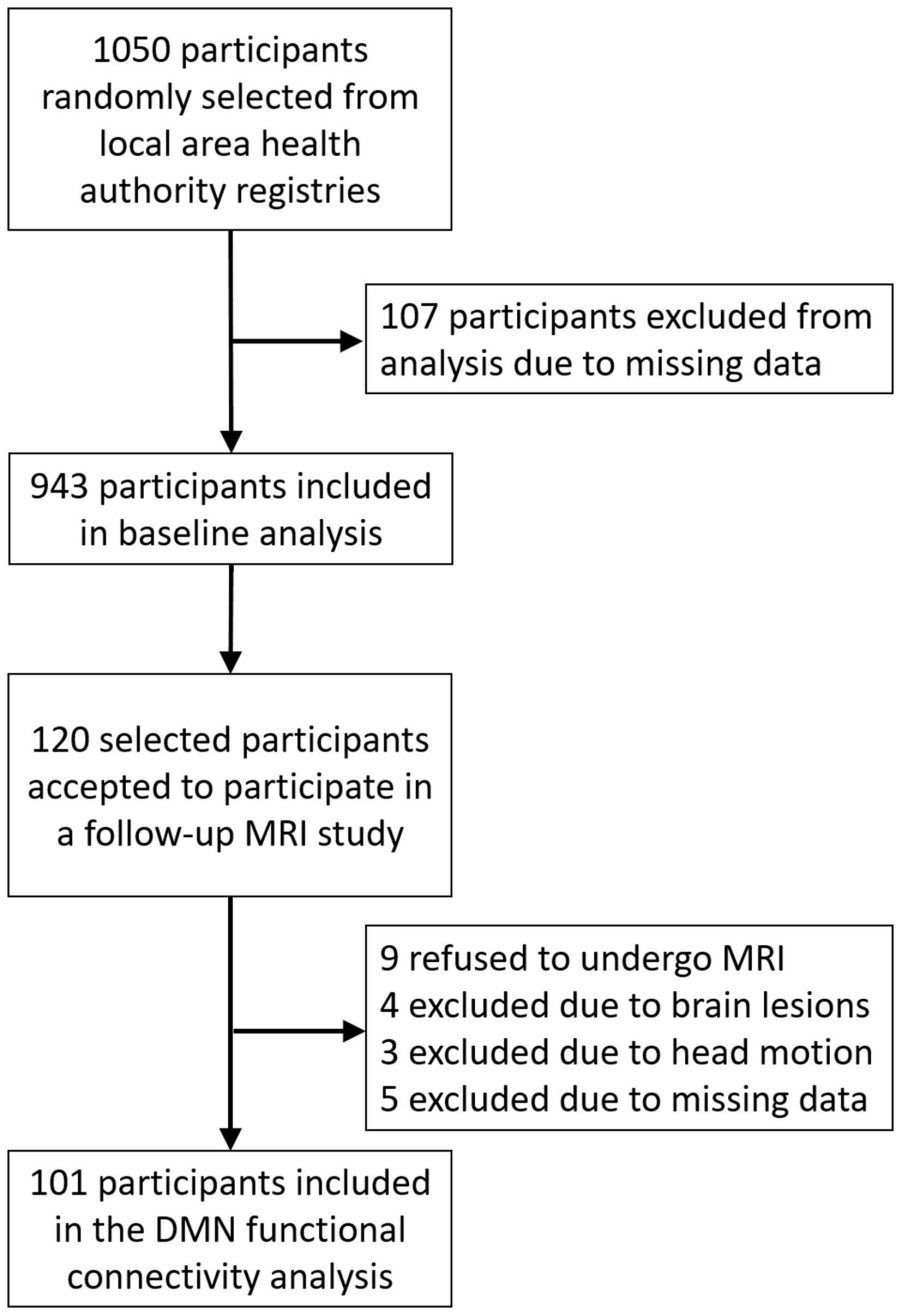
Flow diagram of recruitment and study procedure.

The cohort was composed of 1,051 participants randomly selected from two cities in the north of Portugal (Guimarães and Vizela) using the local area health authority registries as described elsewhere (Santos et al., 2013, 2014) (Figure 1). The cohort is representative of the older Portuguese population with respect to gender (females, *n* = 560; 53.3%) and age (range: 50–97 years; *M* = 67.2, *SD* = 9.24). All the participants were local community-dwellers. Most were retired (*n* = 763, females 51.8%) and located in the medium socio-economic stratum of the Graffar scale (Class III; 61.6%, females 47.3%). For age and gender, the distribution of this database differs in <2% of that of the distribution for the Portuguese population, as estimated by the Portuguese authority on statistics (the “Instituto Nacional de Estatística”) (Instituto Nacional de Estatística IP, 2011). Exclusion criteria included the inability to understand informed consent, participant choice to withdraw from the study, inability to attend the clinical and neuropsychological assessment session(s), dementia and/or diagnosed neuropsychiatric, and/or neurodegenerative disorder (medical records). A team of experienced clinicians performed a standardized clinical interview, which also determined current medication use and was designed to detect and exclude disorders of the central nervous system (e.g., epilepsy and neurodegenerative disorders) as well as overt thyroid pathology (Santos et al., 2013, 2014). As shown in Figure 1, 108 participants were excluded due to missing data, leaving a total of 943 participants to be included in the baseline analysis.

From the initial cohort, 120 participants [matched for gender and age—overall “good” cognitive performance (*n* = 60) and overall “poor” performance (*n* = 60) group, based on their, within normal range, neuropsychological testing (including mood)] were selected for a more comprehensive characterization, including a functional MRI (fMRI) session. Of those, nine refused to have an MRI, four were excluded due to brain lesions, three were excluded due to excessive head motion during the scan, and five were excluded due to missing data. Following exclusions, 101 participants ultimately were included in the fMRI analysis. A detailed characterization of both samples is presented in Table 1; Supplementary Table 1.

**Table 1:** Study sample characterization for the participants included in the cross-sectional analysis and fMRI analysis.

		**Cross-sectional analysis**	**fMRI analysis**
		*****n*** = 943**	*****n*** = 101**
Variable (M; SD)	
	Age (years)	67; 9.17	64; 8.46
	GDS score	10.92; 6.38	10.63; 6.63
	BMI (kg/m^2^)	28.42; 4.38	27.9; 3.62
	Waist circumference (cm)	98.87; 10.42	97.4; 9.06
	Fasting glucose (mg/dL)	94.88; 29.5	92.32; 30.13
	HOMA2-IR	1.29; 1.2	1.36; 1.33
	Triglycerides (mg/dL)	123.14; 70.09	132.96; 100.94
	HDL (mg/dL)	54.51; 13.74	53.9; 13.6
	Systolic BP (mmHg)	143.82; 19.76	141.95; 18.03
	Diastolic BP (mmHg)	80.12; 10.3	81.56; 8.27
Gender (n; %)	
	Female	492; 52.2	47; 46.5
	Male	451; 47.8	54; 53.5
Formal education (n; %)	
	4 years or less	792; 84	74; 73.3
	More than 4 years	151; 16	27; 26.7
Smoking status (n; %)	
	Nonsmoker	659; 69.9	66; 65.3
	Former smoker	222; 23.5	26; 25.7
	Smoker	62; 6.6	9; 8.9
Alcohol consumption (n; %)	
	None	279; 29.6	30; 29.7
	≤ 50 g/day	441; 46.8	41; 40.6
	> 50g/day	223; 23.6	30; 29.7
Physical activity (n; %)	
	None	593; 62.9	68; 67.3
	≤ 3 times/week	143; 15.2	12; 11.9
	>3 times/week	207; 21.9	21; 20.8

### Metabolic and Mood Evaluation

The participants were presented in the morning after overnight fasting and underwent a standardized evaluation that included medical history, anthropometric assessment, blood collection, and blood pressure measurements. The anthropometric measures included weight (Kg), height (m), and abdominal perimeter (cm). Weight and height were subsequently used to calculate body mass index (BMI). Fasting blood glucose, fasting insulin, triglycerides, and high-density lipoproteins were measured using standard methods in a certified laboratory. Blood pressure was assessed three times during the evaluation and the mean value was used. The Geriatric Depression Scale (GDS, long-version) was used to assess mood.

### Resting State fMRI Data Acquisition, Preprocessing and Identification of DMN

The participants were scanned on a clinically approved Siemens Magnetom Avanto 1.5 T (Siemens Medical Solutions, Erlangen, Germany) MRI scanner in Hospital de Braga using a Siemens 12-channel receive-only head coil. During the resting-state fMRI acquisition, using gradient echo-weighted echo-planar images (EPIs), the participants were instructed to keep their eyes closed and to attempt to think about nothing. The imaging parameters were: 180 volumes, Repetition Time (TR) = 2s, Echo Time (TE) = 30 ms, Flip Angle (FA) = 90°, in-plane resolution = 3.5 × 3.5 mm^2^, 30 interleaved slices, slice thickness = 4 mm, imaging matrix 64 × 64, and field of view (FOV) = 224 mm. T1-weighted structural images for anatomical reference were obtained using a magnetization-prepared rapid acquisition by gradient echo (MPRAGE) sequence with voxel resolution 1.0 × 1.0 × 1.0 mm, FoV = 234 × 234 mm^2^, FA of 7°, 176 slices, and TE/TR of 3.48/2730 ms. Before any data processing and analysis were undertaken, all acquisitions were inspected by an experienced neuroradiologist who confirmed that they were not affected by critical head motion and that participants had no brain lesions.

Preprocessing of fMRI data was done using FMRIB Software Library (FSL v5.07) tools. The first five volumes of the acquisition were removed to exclude possible magnetic field inhomogeneities. After this, the data underwent slice timing correction followed by head motion correction. Next, motion scrubbing (Power et al., 2012) was performed to identify and further exclude time-points where head motion could be critical. For motion scrubbing, the standard FSL suggested parameters (REFRMS) and the standard thresholds of exceeding the 75th percentile + 1.5 times the InterQuartile Range were used, meaning that individuals presenting more than 15 motion-contaminated time-points would be excluded. Data on the number of motion-contaminated time-points by subject is presented in Supplementary Figure 1. The functional dataset of each subject was then normalized to Montreal Neurological Institute (MNI) standard space through a procedure that included: (i) skull stripping of the mean image of the functional acquisition and of the structural acquisition, allowing the isolation of brain signal; (ii) rigid-body registration of the mean functional image to the skull-stripped structural scan; (iii) affine registration of the structural scan to the MNI T1 template; (iv) non-linear registration of the structural scan to the MNI T1 template using the affine transformation previously estimated as the initial alignment; (v) nonlinear transformation of the functional acquisition to MNI standard space through the sequential application of the rigid-body transformation and non-linear warp, followed by resampling to 2 mm isotropic voxel size. On the final step, a linear regression of motion parameters, mean white mater and cerebrospinal fluid signal, and motion outliers was performed, and the residuals of the regression were smoothed using a Gaussian kernel smoother with a full width at half maximum of 6 mm (σ = 2.55 mm), were band-pass temporal filtered (0.01–0.08Hz), and were then used for the subsequent analysis.

Probabilistic independent component analysis (PICA) was performed with Multivariate Exploratory Linear Optimized Decomposition into Independent Components (MELODIC), distributed with FSL. ICA is a data driven analysis that isolates components or non-overlapping spatial maps corresponding to regions that manifest coherent time-courses. The software estimated group-wise spatial maps that correspond primarily to Resting State Networks (RSNs) and automatically estimated the number of independent components. A dual regression analysis was used to study subject-specific components.

### Statistical Analysis

Data are presented in mean (M) and standard deviation (SD) for continuous variables and in frequency (*n*) and percentage (%) for categorical variables. Pearson correlations were calculated to measure the strength of the association between the quantitative variables studied. Structural equation models (SEM) were used to model metabolic dysfunction in a fashion similar to that reported by Shen et al. (2003), using MPlus software version 7. The MLR estimator (maximum likelihood parameter estimates with standard errors and a chi-square test statistic that are robust to non-normality and non-independence of observations) was used for parameter estimations and tests of significance. Model fit was assessed using χ^2^, comparative fit index (CFI), Tucker-Lewis index (TLI), and root mean square error of approximation (RMSEA). To determine the cross-sectional associations of the GDS score with each of the composite scores previously calculated (metabolic dysfunction, obesity, glucose dysmetabolism, lipids imbalance, and blood pressure), Multiple Linear Regression Models (MLRM) were performed controlling for age, gender, years of formal education, smoking habits, alcohol consumption, and physical activity. Besides regression coefficients (and confidence intervals), betas and measures of model fit (R^2^, ${\text{R}}_{\text{adjusted}}^{2}$) are also presented. The interaction of age, and metabolic dysfunction (including components) with GDS score was tested using PROCESS 3.0 for IBM SPSS Statistics v25, adjusted for the variables above mentioned. Heatmap plots were obtained using the syntax provided on PROCESS output.

DMN-FC testing was performed using the second-level random effect analyses in SPM12. Multiple regression analysis was performed, and results were considered significant at *p* < 0.001, corrected for multiple comparisons using cluster correction (minimum cluster size of 70 voxels). Minimum cluster size was estimated using 3DClustSim (https://afni.nimh.nih.gov/; AFNI version 17.0.13; National Institute of Mental Health) with the DMN template mask and a significance level of 0.05 (http://findlab.stanford.edu/functional_ROIs.html). Anatomical labeling was performed by a combination of visual inspection and anatomical automatic labeling (AAL) atlas (Tzourio-Mazoyer et al., 2002).

Several independent multiple regressions were performed to test the effect of metabolic dysfunction (or components), the interaction of metabolic dysfunction (or components) with age, the interaction of metabolic dysfunction (or components) with GDS score, and the interaction of metabolic dysfunction (or components) with age and GDS score. The requirements for multiple linear regression analysis were met and the variables were mean-centered to avoid multicollinearity issues. Age, gender, and GDS score were used as covariables when not used as interest variables. For each participant, the mean Z score of the clusters with significant interactions were extracted and the values were used to generate heat maps that allowed visualization of the interaction of the moderator with the association between the focal predictor and the dependent variable.

## Results

### Characterization of Participants

Demographic, metabolic, and lifestyle characterization of participants are presented in Table 1. Information regarding lifestyle variables is presented in the Supplementary Material. Ages of participants ranged from 50 to 97 years (*M* = 67 years and *SD* = 9.17, 47.8% females). The exclusion of the 107 participants with missing data did not significantly change the composition of the original cohort.

Information on the prevalence of significant depressive symptoms and metabolic risk factors according to the IDF classification for MetS (Alberti et al., 2005) are described in Supplementary Material. Bivariate correlations between GDS score and metabolic parameters are presented in Supplementary Table 2. GDS score was significantly correlated with BMI (*r* = 0.106, *p* < 0.01), but not with the other parameters. Metabolic parameters were significantly associated among themselves, with the exception of systolic blood pressure with HDL cholesterol, and diastolic blood pressure with fasting glucose and HDL cholesterol.

### Modeling of Metabolic Dysfunction and Components

**Figure 2:**
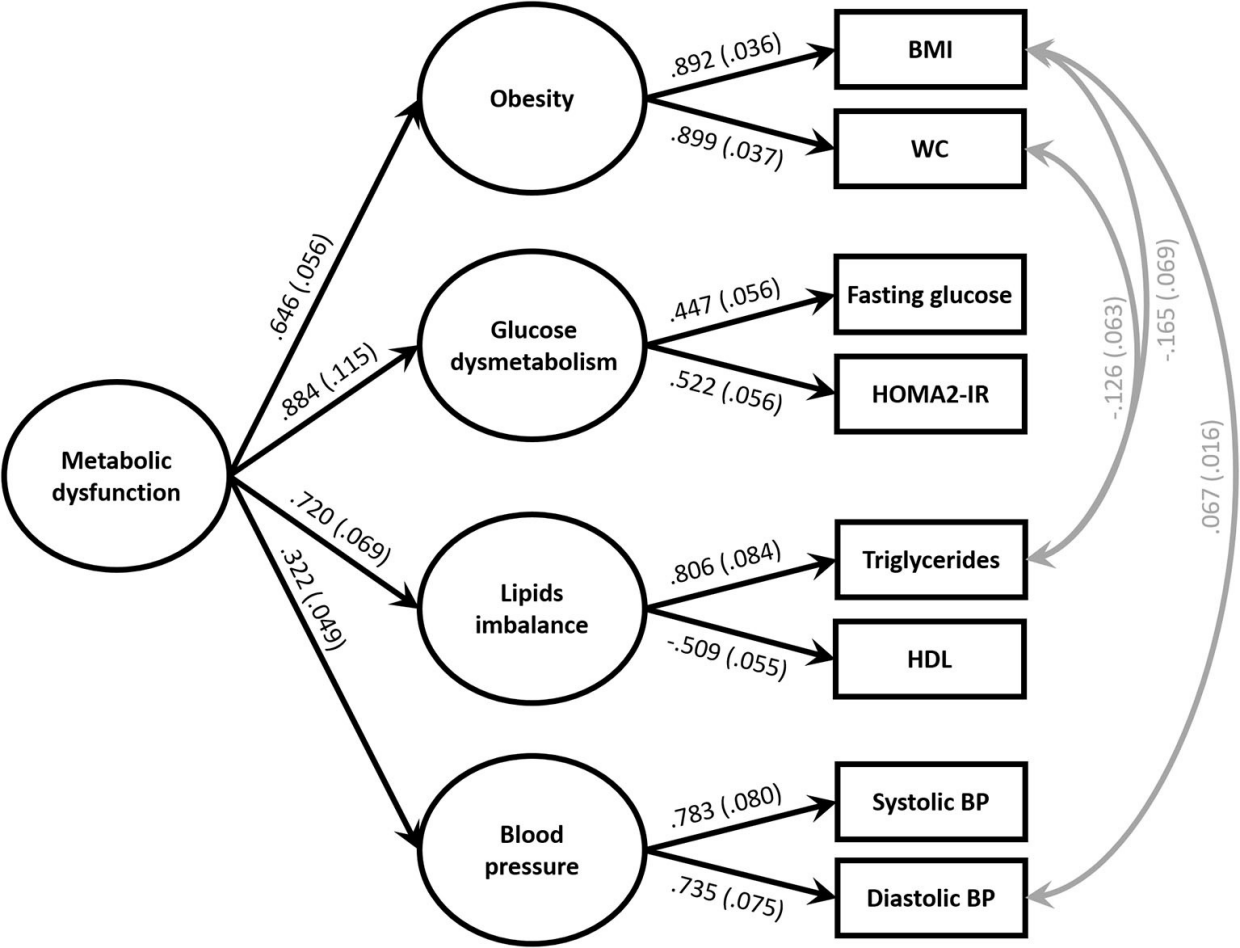
Model of the metabolic dysfunction at baseline. $\chi_{\left(13\right)}^{2}$ = 40.762; *p* < 0.001, comparative fit index (CFI) = 0.981, Tucker Lewis Index (TLI) = 0.958 and root mean square error approximation (RMSEA) = 0.048; *p* = 0.564. Standardized parameter estimates representing factor loadings are shown on paths. All coefficients are significant at *p* > 0.01 for the two-tailed test. To maintain presentation clarity, residual terms are not shown. BMI, body mass index; WC, waist circumference; HDL, high density lipoprotein; BP, blood pressure.

Metabolic dysfunction was modeled as a second-order latent variable similar to the procedure used in Shen et al. (2003, 2006) and Levin et al. (2014) (Figure 2). All indicators were treated as continuous variables. First-order latent variables (obesity, glucose dysmetabolism, lipids imbalance, and blood pressure) were measured by their respective indicators. Specifically, obesity was measured by BMI and waist circumference (WC), glucose dysmetabolism by fasting glucose and HOMA-IR, triglycerides and HDL cholesterol were the indicators for lipid imbalance and systolic and diastolic blood pressure were the measures of blood pressure. A higher-order latent variable, metabolic dysfunction, was created with the first-order latent variables enumerated above.

Three pairs of residual variances were correlated, first between BMI and triglycerides, second between WC and triglycerides, and third between BMI and diastolic blood pressure. The incorporation of these correlations was justified by the known influence of obesity upon triglycerides and blood pressure.

The fit of the model was confirmed by CFI = 0.981, TLI = 0.958 and RMSEA = 0.048. The model demonstrated that metabolic dysfunction could be summarized by four components defined by metabolic risk factors. Metabolic dysfunction was strongly a function of glucose dysmetabolism, moderately of lipid imbalance and obesity, and modestly of blood pressure. Specifically, metabolic dysfunction was explained by glucose dysmetabolism (78%, *p* < 0.001), lipid imbalance (52%, *p* < 0.001), obesity (42%, *p* < 0.001), and blood pressure (10%, *p* = 0.001).

### Associations of Metabolic Dysfunction and Components With Mood Across Later Life

**Figure 3:**
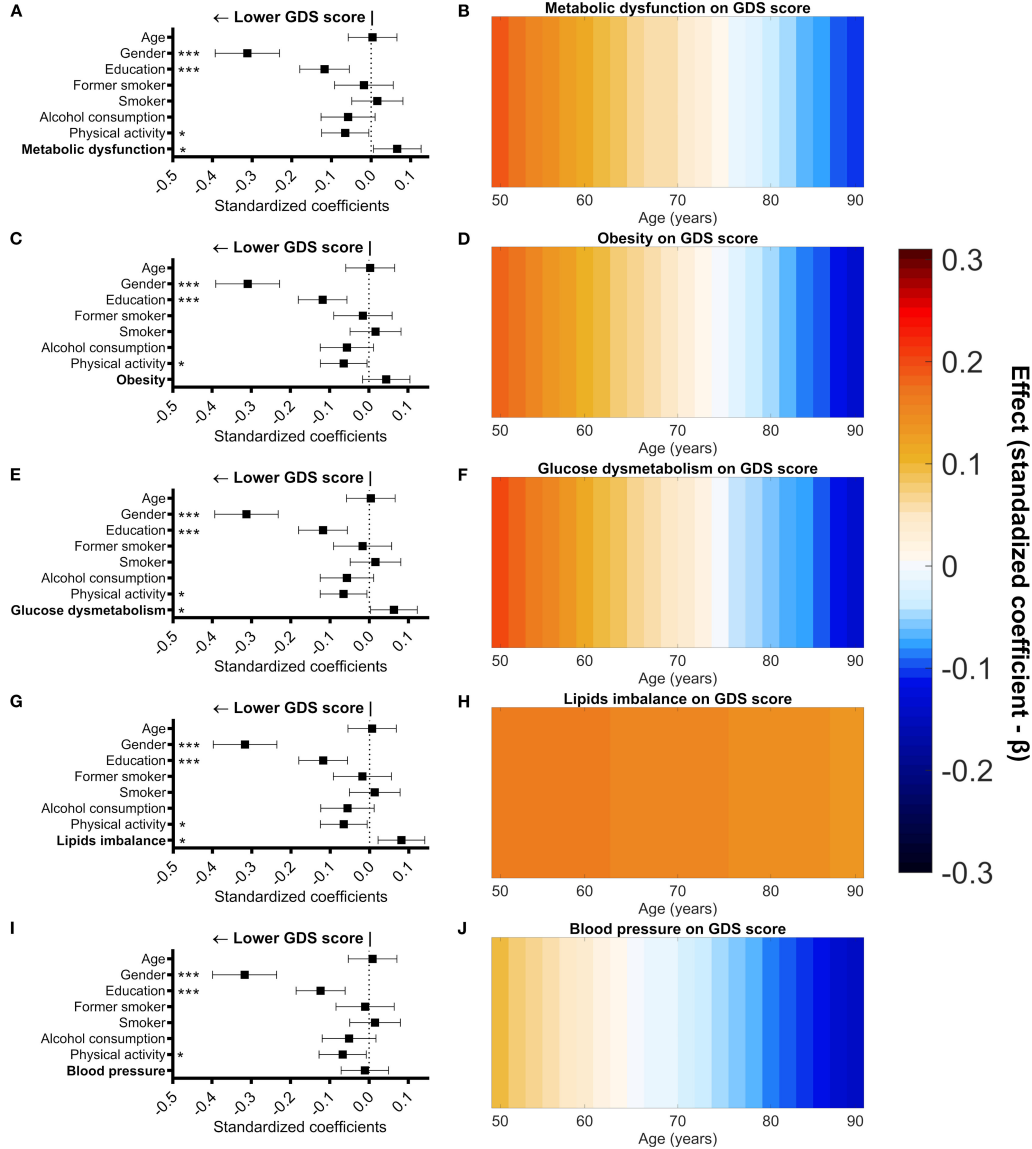
Association of metabolic dysfunction (and components) with GDS score controlled for age, gender, education, smoking status, alcohol consumption, and physical activity. Graphical representation of standardized coefficients (standardized beta) and respective confidence intervals for the independent variables used in the multiple regression linear models **(A,C,E,G,I)**. Male gender, higher formal education (measured in school years), higher physical activity, and lower metabolic dysfunction score were significantly associated with a lower score in the GDS **(A)**. Male gender, higher formal education, and higher physical activity were significantly associated with a lower score in the GDS **(C,I)**. Male gender, higher formal education, higher physical activity, and lower glucose dysmetabolism score were significantly associated with a lower score in the GDS **(E)**. Male gender, higher formal education (measured in school years), higher physical activity, and lower lipids imbalance score were significantly associated with a lower score in the GDS **(G)**. Graphical representation (heat maps) of the variation of standardized coefficients (standardized beta) for the association between metabolic dysfunction (and components) with GDS score across age **(B,D,F,H,J)**. Warm colors represent a positive association and cool colors represent a negative association. A positive association between GDS score and metabolic dysfunction or obesity or glucose dysmetabolism was observed in the younger individuals of our sample and became negative at the older ages **(B,D,F)**. The association between GDS score and lipids imbalance was always positive across the age range **(H)**. No association between GDS score and blood pressure was observed across the age range **(J)**. Gender (0 = females; 1 = males), smoking (reference—non-smoker), alcohol consumption (g/day), and physical activity (0 = sedentary; 1 = <3 times/week; 2 = over 3 times/week). *p < 0.05; **p < 0.01; ***p < 0.001.

To test the association of metabolic dysfunction and its components with depressive mood, MLRM (Figures 3A,C,E,G,I; Supplementary Table 3) was used. GDS score was used as dependent variable, while metabolic dysfunction and its components as independent variable in different models, controlling for age, gender, education, smoking status, alcohol consumption, and physical activity. All the models significantly predicted the GDS score (*p* < 0.001) and explained approximately 18% (*R*^2^ = 0.179 to 0.185) of the variance of the GDS score.

In all the models, male gender, higher education (>4 years of formal education), alcohol consumption, and physical activity higher than three times per week were significantly associated with a lower GDS score.

In the respective models, metabolic dysfunction (β = 0.066, *p* = 0.029), glucose dysmetabolism (β = 0.062, *p* = 0.039), and lipids imbalance (β = 0.076, *p* = 0.011) were significantly associated with a higher GDS score. Furthermore, no linear association between depressive mood and obesity was observed, but a significant association between depressive mood and the quadratic term for obesity (Obsety^2^) was observed (β = 0.081, *p* = 0.007—Supplementary Table 4).

The effect of age on the association between metabolic dysfunction (or its components) and mood was tested through moderation analysis in MLRM (Figures 3B,D,F,H,J; Supplementary Table 3). A significant moderation effect of age was observed in the association of GDS score with metabolic dysfunction (metabolic dysfunction x age—β = −0.096, *p* = 0.047), obesity (obesity x age—β = −0.065, *p* = 0.032), and glucose dysmetabolism (glucose dysmetabolism x age—β= −0.066, *p* = 0.028). This moderating effect of age reflects a positive correlation between the measured variables in younger participants, but one which diminishes with advancing age. The association of the quadratic term of obesity with the GDS score (Supplementary Table 4) was not influenced by age.

The association between GDS score and the lipids imbalance component was not moderated by age (lipids imbalance x age—β= −0.017, *p* = 0.592) indicating that lipids imbalance is positively associated with GDS score regardless of age. Also, no moderating effect of age was observed upon the association of blood pressure and GDS score (blood pressure x age—β= −0.050, *p* = 0.098), indicating that blood pressure and mood are not significantly associated with one another at any age in the study population.

### Impact of Metabolic Dysfunction (and Components) in the Association Between GDS Score and DMN FC

**Figure 4:**
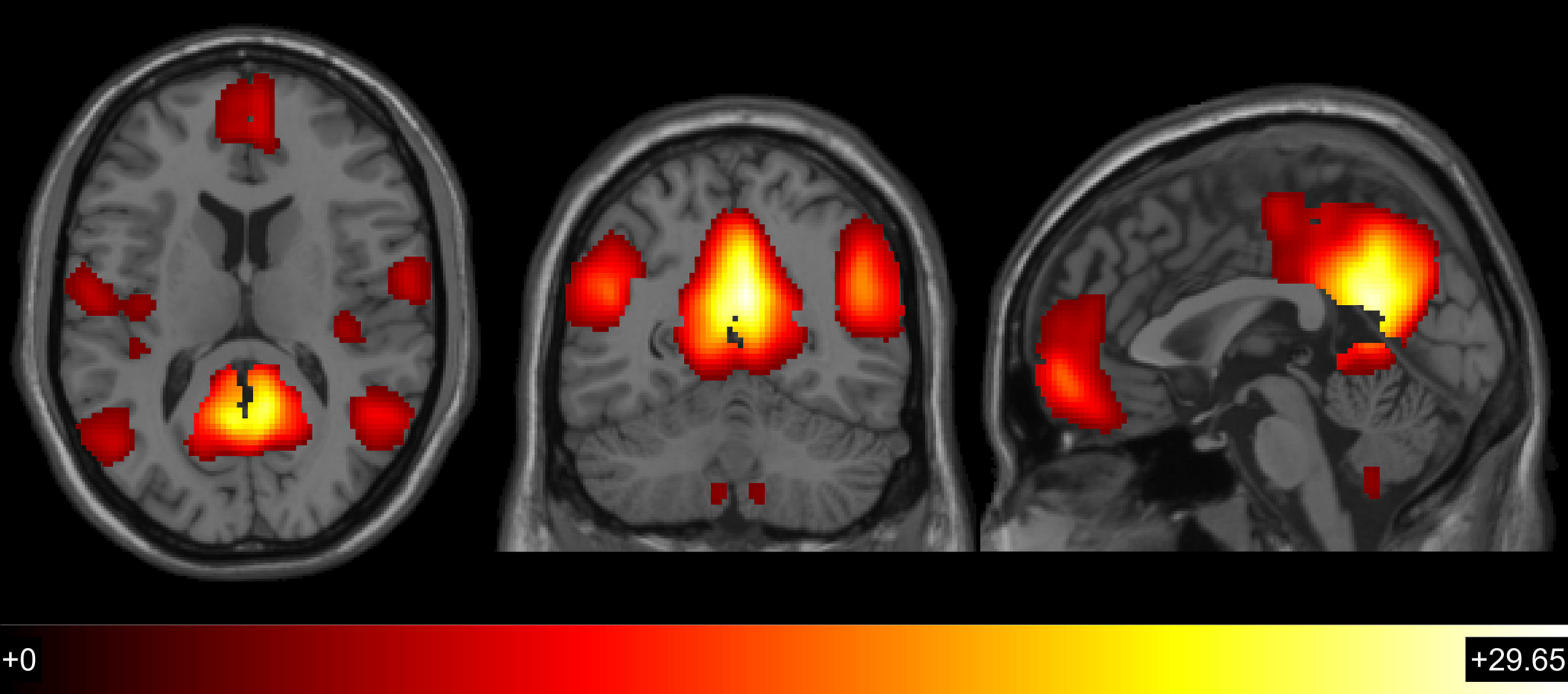
Global patterns of default mode network functional connectivity (*t* > 3.1667; *p* <0.001; *df* = 109).

First, the pattern of FC of the classical DMN regions during the resting state was visually confirmed (Figure 4). The effects of metabolic dysfunction (or its components) and the interaction of age with metabolic dysfunction (or components) in the DMN-FC were evaluated, in independent MLRM, and the results did not survive to the significance threshold here employed.

**Figure 5:**
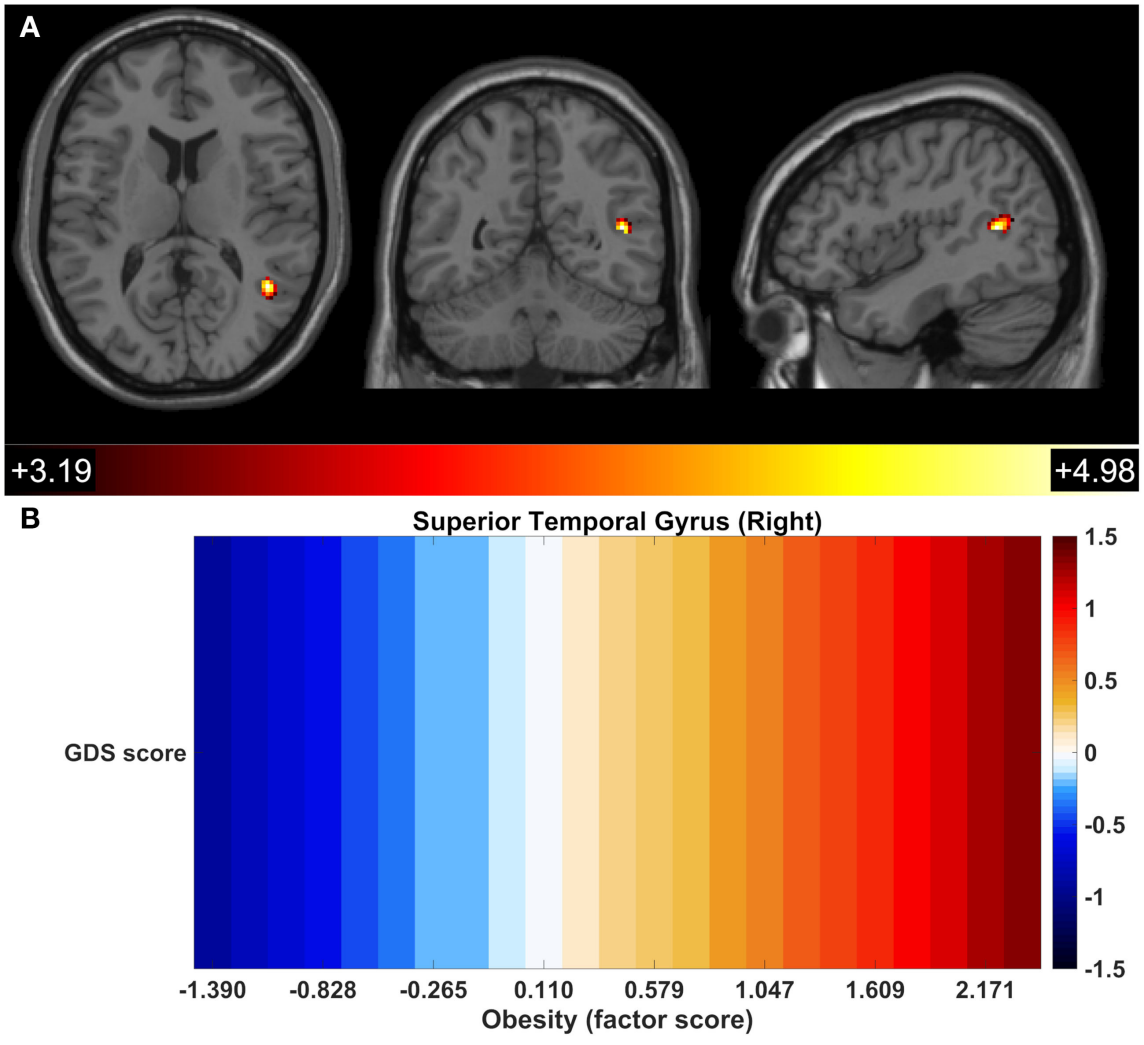
Functional connectivity between a focal region of the right superior temporal gyrus and the other components of the DMN reflected GDS-obesity interaction. DMN regions presenting significant interactions of GDS x Obesity **(A)**. Association of the GDS score with the FC of the right superior temporal gyrus across obesity score; colors represent standardized coefficients (β) **(B)**.

Next, the moderating effect of metabolic dysfunction (and its components) in the association between GDS score and DMN-FC was tested. An interaction between obesity and GDS score was observed in the right superior temporal gyrus (Figure 5A; Table 2). The FC of the right superior temporal gyrus increased with higher GDS score in the participants that have higher obesity scores. Individuals with lower obesity scores, however, manifest decreased FC with increasing GDS (Figure 5B). No other interaction between metabolic dysfunction or its components with GDS was significant at the defined threshold.

**Table 2:** Effect of the interaction between GDS x obesity and between age x GDS x blood pressure on the default mode network FC (multiple regressions, cluster correction, *p* <0.001).

**Effect**	**Region**	**Peak MNIcoordinates**	**Cluster size (voxels)**	**MaximumZ score**
GDS x Obesity	Superior Temporal Gyrus (Right)	44, −54, 12	81	4.98
Age x GDS x Blood pressure	Supramarginal Gyrus (Right)	60, −50, 24	234	4.19

### The Moderating Effect of Age Upon the Interaction Between Metabolic Dysfunction and GDS Score With Respect to DMN-FC

**Figure 6:**
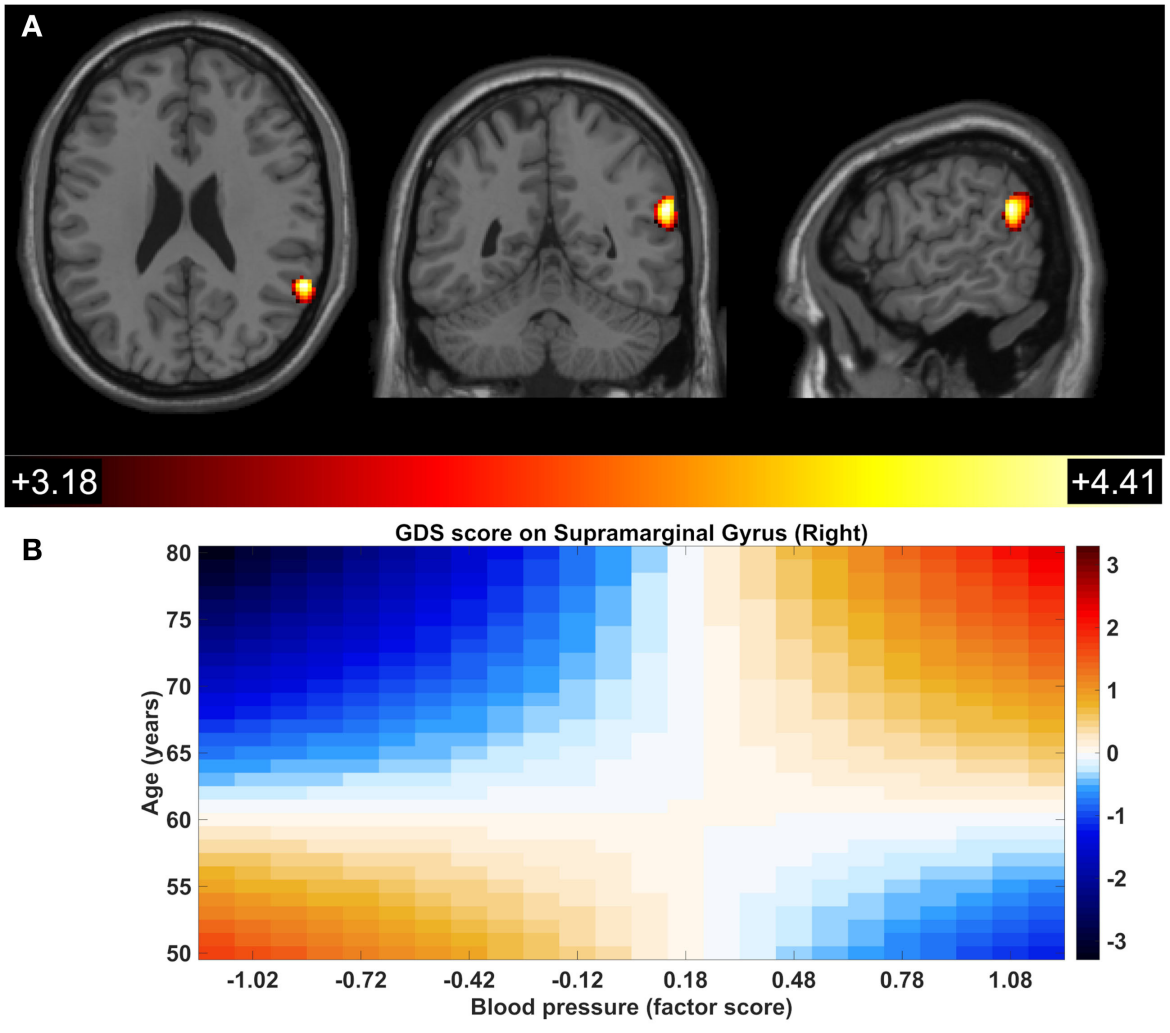
Significant age x GDS x blood pressure interaction within the default mode network in the right supramarginal gyrus. Colors represent standardized coefficients (β). DMN regions presenting significant interactions of age x GDS x blood pressure **(A)**. Association of the GDS score with the FC of the right supramarginal gyrus across age and blood pressure score; colors represent standardized coefficients (β) **(B)**.

The influence of age on the interaction of metabolic dysfunction with GDS score upon DMN-FC was also assessed. A significant moderating effect of age was observed on the interaction of blood pressure with the GDS score in the level of FC of the right supramarginal gyrus with the other components of the DMN (Figure 6A; Table 2). For the younger participants (mean age −1 SD), the FC of the right supramarginal gyrus with the DMN increased with higher GDS score in those with lower blood pressure (mean blood pressure −1 SD) (conditional effect, *B* = 0.021, *p* = 0.02), but was not significant for those with higher blood pressure (mean blood pressure + 1 SD) (conditional effect, B = −0.009, *p* = 0.359). Also, in the group of older participants (mean age + 1 SD), a decrease in the FC accompanied the GDS score increase in those with lower blood pressure (mean blood pressure −1 SD) (conditional effect, *B* = −0.054, *p* < 0.001), while the opposite pattern was observed for those with higher blood pressure (mean blood pressure + 1 SD) (conditional effect, *B* = 0.025, *p* = 0.012) (Figure 6B). No other moderating effect of age upon the interactions of metabolic dysfunction and components with GDS score was statistically significant.

## Discussion

In this study, the association between metabolic dysfunction and depressive mood in community-dwellers aged 50 years and older was investigated, and the relationship between those factors with DMN-FC. This study showed that depressive mood is linearly associated with metabolic dysfunction, glucose dysmetabolism, and lipid imbalance. Conversely, the association of obesity with depressive symptomatology was U-shaped. The influence of age upon the strength of the association of depressive mood with metabolic dysfunction, obesity, and glucose dysmetabolism was substantial. Of notice, obesity can modify the association of GDS score with FC in the DMN, and the interaction of age with blood pressure also affect the association of GDS score with the FC in the DMN.

Glucose dysmetabolism appears to be the essential feature of metabolic dysfunction, in accordance with other cross-sectional and longitudinal models (Shen et al., 2003, 2006). Insulin resistance is the most widely accepted hypothesis for the pathophysiology of the MetS (Eckel et al., 2005), which would tend to support the large contribution of glucose dysmetabolism to metabolic dysfunction. The blood pressure component made a significant weaker contribution to metabolic dysfunction in all models. Shen et al. (2003) reported a similar finding and argued that blood pressure may be related to metabolic dysfunction only secondarily.

As expected, it was observed that metabolic dysfunction was associated with depressive mood. However, the analytic strategy of the study does not allow inference about the direction of this association. In a meta-analysis, Pan et al. (2012) observed that baseline depression predicts the risk of MetS and baseline MetS predicts the risk of depression, indicating a bidirectional association. Since MetS is a constellation of metabolic abnormalities, the association of MetS with depressive mood is likely to be mediated by its components. As previously stated, age plays an important role in moderating the association between metabolic dysfunction and depressive mood. It was shown that the expression of MetS varies with age and that different combinations of MetS components are differentially associated with mortality risk (Kuk and Ardern, 2010). It is also possible that the age variation in the expression of metabolic dysfunction influences its association with depressive mood.

There is strong evidence for a bidirectional association between obesity and mood disorders (Luppino et al., 2010), but, a linear association between obesity and GDS score was not observed. One possible explanation may be reflected in the U-shaped association between BMI and depressive mood (de Wit et al., 2009). In fact, a significant association of depressive mood with the quadratic term of the obesity factor was observed, which is consistent with a U-shaped relationship. Accumulating evidence suggests that obesity and depression may mutually influence and reinforce one another (Taylor and Macqueen, 2010; Mansur et al., 2015; Lee et al., 2016). The observed effect of age upon the association between depressive mood and obesity could potentially mask a linear association between them. In the present study, it was observed that an association of obesity with depressive mood was positive for the younger participants and vanished with increasing age. Low BMI in older age can be a surrogate marker of chronic illness (illness-related weight loss) and it is well-established that late-life depression frequently occurs in the context of medical illness (Fiske et al., 2009; Flegal et al., 2011). Furthermore, it has previously been hypothesized that higher BMI could indicate a greater physiologic and functional reserve (due to higher muscle mass), which then protects against depressive mood in later life (Ho et al., 2008).

A significant interaction between obesity and GDS score with the FC of the right superior temporal gyrus with the other identified components of DMN was also observed. The FC of the right superior temporal gyrus was positively associated with GDS score in individuals with the higher obesity scores. Conversely, an opposite effect was observed for the individuals with lower obesity scores. In older adults, higher BMI has been associated with a decrease in FC of the posterior DMN (Beyer et al., 2017). DMN activity and connectivity has been demonstrated to be involved in one's own thoughts and feelings, self-referential thinking, recall the past, and in planning for the future. One possible explanation for the hyperactivity and connectivity of DMN in patients with depression might be that it represents an inability to navigate away from their internal emotional states (Whitfield-Gabrieli and Ford, 2012; Kaiser et al., 2015). The results presented in this study are suggestive of a more resilient pattern of FC within the DMN in older individuals with low obesity score.

Another endocrine mediator associated with depressive mood is insulin resistance (Lustman et al., 2000; Kan et al., 2013). Data from epidemiological studies indicate that depression is twice as common among those with diabetes than in the general population, and that having diabetes doubles the risk of depression (Anderson et al., 2001). Furthermore, it seems that while the association is bidirectional, it is stronger in the direction of depression to type 2 diabetes (Mezuk et al., 2008). Here, a positive correlation between glucose dysmetabolism and depressive mood was observed. Depression is associated with the activation of the HPA axis and production of pro-inflammatory cytokines, which can induce insulin resistance (Silva et al., 2012; Yokoyama et al., 2015). Another hypothesis put forward to explain this association is that the inadequate glucose utilization that results from central insulin resistance is responsible for change at the neuronal level in vulnerable brain regions (e.g., limbic system) observed in patients with depressive disorders (Rasgon and Kenna, 2005). Data from animal models show that brain-specific knockout of insulin receptor (NIRKO) in mice promotes age-related anxiety and depressive-like behavior through an alteration in dopamine turnover (Kleinridders et al., 2015). The relationship between depressive mood and glucose dysmetabolism was significantly moderated by age, similar to that was observed for metabolic dysfunction and obesity. In younger participants, the association was positive but lost strength with increasing age. A reasonable explanation for such a pattern is not evident, but it is recognized that selection bias may be present. It is more likely that older individuals with higher levels of depression and higher comorbidities refused to participate in the study.

A significant positive association between depressive symptoms and lipid metabolism which was not moderated by age was also observed. Greater lipid imbalance factor is manifest in higher values of triglycerides and lower levels of HDL cholesterol. Research on the association between serum lipids and depression has generated conflicting results and has focused primarily on total cholesterol (Beydoun et al., 2015). Lower HDL cholesterol was reported to be associated with depression (Sagud et al., 2009; Kim et al., 2011). While high triglyceride levels were found in patients with bipolar depression when compared with healthy controls (Sagud et al., 2009). In persistent-severe depression, the odds ratio for low HDL cholesterol and hypertriglyceridemia were significantly increased in males, and a similar association was observed for women with respect to hypertriglyceridemia (Kim et al., 2015). Higher levels of triglycerides and lower HDL cholesterol in ongoing major depression, compared to remitted depression and controls, also has been reported (van Reedt Dortland et al., 2010).

Blood pressure was the only component of metabolic dysfunction, which was not linked to depressive mood, as seen by others. The association of blood pressure with depression is controversial. Some cross-sectional studies reported an association between depression and low blood pressure (Hildrum et al., 2007; Lenoir et al., 2008), while other longitudinal studies found that depressive symptoms predicted low blood pressure (Hildrum et al., 2008) and that low blood pressure was a risk factor for higher levels of depression (Paterniti et al., 2000). Other publications reported a significant association of late-life depression with hypertension (Lavretsky et al., 1998). Additional studies are therefore needed to test the Vascular Depression hypothesis (Taylor et al., 2013), which posits that cerebrovascular disease, of which hypertension is one element, may predispose, precipitate, or perpetuate certain geriatric depressive syndromes.

Interestingly, a significant interaction between blood pressure, age, and GDS score was observed in the FC of the supramarginal gyrus. Recently, Gu et al. (2019) reported that, compared to controls, hypertensive patients with normal cognition manifested increased FC in the core subsystem of the DMN, including that of the right supramarginal gyrus. Furthermore, Zhang et al. (2011) found increased node centrality in drug-naive, first-episode major depressive disorder patients in components of the DMN, including the right supramarginal gyrus. Similarly, decreased FC in the DMN has been widely reported in older subjects (Andreescu et al., 2011; Wu et al., 2011; Soares et al., 2017). Collectively, these multiple studies suggest that the pattern of the FC of the right supramarginal gyrus is complex and is influenced by multiple factors.

This study is original in the use of structural equation models to explore the association of metabolic dysfunction with depressive mood, the impact of age upon those associations, and in the combined influence of those multiple factors upon connectivity patterns in the DMN. The age composition of the sample is representative of the Portuguese population and represents a strength of this study. A number of reports have addressed the association of MetS and depressive mood (Pan et al., 2012). It is important to refer that the MetS is not uniformly classified, which likely justifies discrepant findings (Kuk and Ardern, 2010). It is important to acknowledge that medication usage and duration may impact on DMN-FC (Yan et al., 2019). In this study, the impact of medication in the DMN-FC was not controlled and therefore the results should be interpreted with caution. Another limitation, inherent to the use of the IDF classification for MetS, is that it excludes individuals who have metabolic risk factors but not central obesity (Oda, 2012). To overcome those limitations, structural equation modeling was used, more specifically confirmatory factor analysis, to replicate the hierarchical four-factor model first proposed by Shen et al. (2003, 2006) and later replicated by Levin et al. (2014). The use of structural equation modeling allowed to consider the separate contribution of each component to metabolic dysfunction. Additionally, it allows the use of continuous variables rather than employing a dichotomous classification, which relies upon a proxy to assess severity of metabolic dysfunction. As a measure of depressive mood, the GDS that measures depressive symptomatology rather than depression was used. The inclusion of subjects that have a finite GDS score but are not clearly depressed can weaken the association observed here, yet there is evidence that in older adults, even depressive symptoms are associated with adverse outcomes and morbidity (Meeks et al., 2011). The analysis of the moderating effects on the FC of the DMN adds to the analysis of the association between mood and metabolic dysfunction by exploring the central nervous system impact of peripheral mechanisms. Better understanding of the complex associations between metabolic dysfunction and depressive mood will be dependent upon detailed analysis of the multiple interactions between the variables studied here.

## Data Availability Statement

The raw data supporting the conclusions of this article will be made available by the authors, without undue reservation.

## Ethics Statement

The studies involving human participants were reviewed and approved by Portuguese national ethical committee (Comissão Nacional de Proteção de Dados) and by the local ethics review boards (Hospital de Braga, Braga; Centro Hospitalar do Alto Ave, Guimarães). The patients/participants provided their written informed consent to participate in this study.

## Author Contributions

CP-N, JR, PM, PCo, and JB performed the statistical analysis. CP-N, JR, PM, TC, LA, RM, PCo, and JB contributed to the data analyses and discussion. NS maintained the database and organized the neurocognitive/psychological sessions. PCu organized the evaluation sessions, participant recruitment and collected the data. CP-N, TC, and LA collected the data. AC, RM, PM, and JS collected and processed fMRI data. CP-N wrote the first draft of the manuscript. JP, NS, and JB conceived and designed the study. NS had access to all the data in the study. All the authors revised the manuscript.

## Conflict of Interest

The authors declare that the research was conducted in the absence of any commercial or financial relationships that could be construed as a potential conflict of interest.

## Publisher's Note

All claims expressed in this article are solely those of the authors and do not necessarily represent those of their affiliated organizations, or those of the publisher, the editors and the reviewers. Any product that may be evaluated in this article, or claim that may be made by its manufacturer, is not guaranteed or endorsed by the publisher.
