# Supplementary material for: The Association of Metabolic Dysfunction and Mood Across Lifespan Interacts With the Default Mode Network Functional Connectivity
Source: Front Aging Neurosci. 2021 Aug 2;13:618623. doi: 10.3389/fnagi.2021.618623 (PMC8364979; doi:10.3389/fnagi.2021.618623)
Supplement: Supplementary file 1 [file Data_Sheet_1.docx]

Supplementary Material

**Methods – Information on lifestyle variables**

For lifestyle, at baseline, alcohol consumption (none, 50 or less, and more than 50 grams/day), physical activity status (none, <3, and over 3 times per week), and smoking habits (non-smoker, former smoker, and current smoker) were considered. Alcohol consumption was calculated and recorded as total grams/day, taking as reference the estimations of grams of alcohol per glass for each consumed beverage. Physical activity included any planned activities (e.g., walking, jogging, swimming) that comprised a continuous 30 min effort (which could range from light, to moderate and vigorous) above the everyday living activities such as the case of regular short walk to the grocery store. Activity quantity rather than intensity was considered due to the mixed clinical profiles and age range of the study population. Alcohol consumption and smoking habits were self-reported by the participants during the clinical interview and were referent to the current habits.

**Results – Additional information on sample characterization**

Approximately half of our sample (n=449, 47.6%) presented some degree of depressive symptomatology (GDS score ≥11). Regarding BMI, 433 subjects (45.9%) presented overweight (25 kg/m^2^ ≤ BMI < 30 kg/m^2^), 300 (31.8%) were obese (BMI ≥ 30 kg/m^2^) and only 210 participants (22.3%) had normal weight (BMI < 25 kg/m^2^). The majority had an increased WC (n=781, 82.8%), that was more frequent in females (females WC > 80 cm - n=477, 97.7%; males WC > 94 cm – n=304, 67.4%) indicating a high risk of metabolic complications among those participants. Fasting plasma glucose was elevate in 245 participants (26%; fasting glucose≥100 mg/dL), raised triglycerides were present in 215 participants (22.8%; triglycerides>150 mg/dL), 223 (23.6%) participants had reduced HDL-cholesterol (HDL-cholesterol<50mg/dL in females and <40mg/dL in males) and elevated blood pressure was present in 702 participants (74.4%: systolic blood preassure≥130mmHg and/or diastolic blood preassure≥85 mmHg).

**Supplementary Table 1.** Age and gender distribution on cross-sectional analysis and fMRI analysis

|  |  | **Cross-sectional analysis** | |  | **fMRI analysis** | |
| --- | --- | --- | --- | --- | --- | --- |
| **Age category (years)** | | **Female (n; %)** | **Male (n; %)** |  | **Female (n; %)** | **Male (n; %)** |
|  | 55 or less | 65; 13.2 | 72; 16 |  | 6; 12.8 | 13; 24.1 |
|  | 56 to 60 | 67; 13.6 | 52; 11.5 |  | 9; 19.1 | 10; 18.5 |
|  | 61 to 65 | 53; 10.8 | 43; 9.5 |  | 5; 10.6 | 6; 14.8 |
|  | 66 to 70 | 119; 24.2 | 101; 22.4 |  | 15; 31.9 | 11; 20.4 |
|  | 71 to 75 | 104; 21.1 | 94; 20.8 |  | 8; 17 | 6; 11.1 |
|  | 76 or more | 84; 17.1 | 89; 19.7 |  | 4; 8.5 | 6; 11.1 |


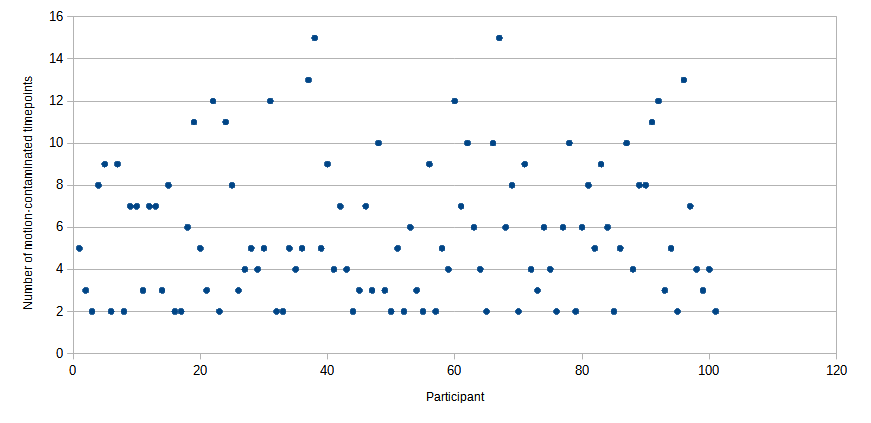


**Supplementary Figure 1.** Number of motion-contaminated time-points by subject.

**Supplementary Table 2.** Correlation matrix among GDS score and metabolic parameters.

|  | **GDS score** | **BMI** | **Waist circ.** | **Fasting glucose** | **HOMA2-IR** | **Triglycerides** | **HDL** | **Systolic BP** | **Diastolic BP** |
| --- | --- | --- | --- | --- | --- | --- | --- | --- | --- |
| **GDS score** | 4.700 | 2.966 | 4.048 | 2.631 | .173 | 17.758 | -.017 | -7.606 | .798 |
| **BMI** | .106** | 19.165 | 36.620 | 25.786 | 1.581 | 52.406 | -11.734 | 1.530 | 8.536 |
| **Waist circ.** | .061 | .803*** | 108.509 | 67.196 | 3.446 | 152.989 | -29.292 | 31.967 | 16.084 |
| **Fasting glucose** | .014 | .200*** | .219*** | 87.047 | 8.241 | 498.476 | -69.498 | 9.018 | 14.575 |
| **HOMA2-IR** | .023 | .302*** | .276*** | .233*** | 1.434 | 2.322 | -3.490 | 2.072 | .849 |
| **Triglycerides** | .040 | .171*** | .210*** | .241*** | .242*** | 4913.080 | -399.321 | 224.146 | 135.484 |
| **HDL** | .000 | -.195*** | -.205*** | -.171*** | -.212*** | -.414*** | 188.908 | -3.264 | -3.715 |
| **Systolic BP** | -.060 | .122*** | .155*** | .154*** | .088** | .162*** | -.012 | 39.304 | 116.095 |
| **Diastolic BP** | .012 | .189*** | .150*** | .048 | .069* | .188*** | -.026 | .570*** | 106.104 |

*p < .05level; **p< .01; ***p< .001. Parameters’ variances are represented on the diagonal of the table (light grey); parameters’ covariances are represented on the upper-triangle (dark grey); correlation coefficients are represented on the lower triangle (no shading).

**Supplementary Table 2.** Linear regression metabolic dysfunction and components in mood and moderation analysis of age.

| **GDS score** |  | | |  |  | | |
| --- | --- | --- | --- | --- | --- | --- | --- |
|  | **B (CI 95%)** | **SE** | **β; *p*** |  | **B (CI 95%)** | **SE** | **β; *p*** |
| Age | .002 (-.041; .046) | .022 | .004;.911 |  | .001 (-.042; .044) | .022 | .001; .968 |
| Gender ^a^ | **-3.988 (-5.022; -2.954)** | **.527** | **-.312; <.001** |  | **-4.072 (-5.108; -3.036)** | **.528** | **-.319; <.001** |
| Formal education ^b^ | **-2.041 (-3.123; -.960)** | **.551** | **-.117; <.001** |  | **-2.043 (-3.122; -.963)** | **.550** | **-.117; <.001** |
| Former smoker ^c^ | -.264 (-1.376; .849) | .567 | -.018; .642 |  | -.270 (-1.381; .84) | .566 | -.018; .633 |
| Smoker ^c^ | .408 (-1.254; 2.070) | .847 | .016; .630 |  | .471 (-1.19; 2.131) | .846 | .018; .578 |
| Alcohol consumption | -.010 (-.023; .002) | .006 | -.058; .096 |  | -.010 (-.022; .003) | .006 | -.054; .120 |
| Physical activity | **-.397 (-.759; -.035)** | **.184** | **-.065; .031** |  | **-.419 (-.78; -.057)** | **.184** | **-.069; .023** |
| Metabolic dysfunction | **.896 (.086; 1.706)** | **.413** | **.066; .030** |  | .804 (-.01; 1.618) | .415 | .059; .053 |
| Metabolic dysfunction X Age | --- | --- | --- |  | **-.096 (-.19; -.001)** | **.048** | **-.060; .047** |
| F_(df1;df2)_; R^2^; R^2^_adjusted_; *p* | 22.884_(8; 934)_; .164; .157; <.001 | | |  | 20.846_(9; 933)_; .167; .159; <.001 | | |
| Age | .002 (-.041; .046) | .022 | .003; .913 |  | .000 (-.044; .043) | .022 | .000; .988 |
| Gender ^a^ | **-3.95 (-4.991; -2.908)** | **.531** | **-.309; <.001** |  | **-4.002 (-5.043; -2.961)** | **.530** | **-.314; <.001** |
| Formal education ^b^ | **-2.053 (-3.138; -.967)** | **.553** | **-.118; <.001** |  | **-2.048 (-3.131; -.964)** | **.552** | **-.118; <.001** |
| Former smoker ^c^ | -.231 (-1.345; .883) | .568 | -.015; .684 |  | -.233 (-1.345; .879) | .567 | -.015; .681 |
| Smoker ^c^ | .437 (-1.228; 2.102) | .848 | .017; .606 |  | .585 (-1.082; 2.251) | .849 | .023; .491 |
| Alcohol consumption | -.010 (-.023; .002) | .006 | -.056; .106 |  | -.010 (-.022; .003) | .006 | -.054; .121 |
| Physical activity | **-.391 (-.754; -.028)** | **.185** | **-.064; .035** |  | **-.422 (-.785; -.059)** | **.185** | **-.069; .023** |
| Obesity | .329 (-.12; .778) | .229 | .044; .151 |  | .367 (-.083; .817) | .229 | .050; .110 |
| Obesity X Age | --- | --- | --- |  | **-.056 (-.106; -.005)** | **.026** | **-.065; .032** |
| F_(df1;df2)_; R^2^; R^2^_adjusted_; *p* | 22.491_(8; 934)_; .162; .154; <.001 | | |  | 20.584_(9; 933)_; .166; .158; <.001 | | |
| Age | .003 (-.040; .046) | .022 | .004; .897 |  | .001 (-.042; .044) | .022 | .002; .958 |
| Gender ^a^ | **-3.999 (-5.033; -2.965)** | **.527** | **-.313; <.001** |  | **-4.083 (-5.118; -3.048)** | **.527** | **-.320; <.001** |
| Formal education ^b^ | **-2.057 (-3.138; -.977)** | **.551** | **-.118; <.001** |  | **-2.045 (-3.123; -.966)** | **.550** | **-.118; <.001** |
| Former smoker ^c^ | -.252 (-1.365; .860) | .567 | -.017; .656 |  | -.267 (-1.378; .843) | .566 | -.018; .636 |
| Smoker ^c^ | .408 (-1.254; 2.071) | .847 | .016; .630 |  | .476 (-1.184; 2.136) | .846 | .018; .574 |
| Alcohol consumption | -.010 (-.023; .002) | .006 | -.057; .101 |  | -.010 (-.022; .003) | .006 | -.053; .125 |
| Physical activity | **-.400 (-.762; -.038)** | **.184** | **-.066; .030** |  | **-.423 (-.785; -.062)** | **.184** | **-.070; .022** |
| Glucose dysmetabolism | **1.149 (.057; 2.24)** | **.556** | **.063; .039** |  | 1.045 (-.048; 2.138) | .557 | .057; .061 |
| Glucose dysmetabolism X Age | --- | --- | --- |  | **-.143 (-.270; -.015)** | **.065** | **-.066; .028** |
| F_(df1;df2)_; R^2^; R^2^_adjusted_; *p* | 22.818_(8; 934)_; .163; .156; <.001 | | |  | 20.902_(9; 933)_; .168; .160; <.001 | | |
| Age | .005 (-.038; .048) | .022 | .007; .835 |  | .004 (-.039; .047) | .022 | .006; .843 |
| Gender ^a^ | **-4.047 (-5.079; -3.015)** | **.526** | **-.317; <.001** |  | **-4.071 (-5.107; -3.035)** | **.528** | **-.319; <.001** |
| Formal education ^b^ | **-2.059 (-3.137; -.981)** | **.549** | **-.118; <.001** |  | **-2.072 (-3.152; -.993)** | **.550** | **-.119; <.001** |
| Former smoker ^c^ | -.272 (-1.382; .838) | .566 | -.018; .631 |  | -.270 (-1.38; .841) | .566 | -.018; .634 |
| Smoker ^c^ | .345 (-1.315; 2.005) | .846 | .013; .684 |  | .348 (-1.313; 2.009) | .846 | .014; .681 |
| Alcohol consumption | -.010 (-.022; .002) | .006 | -.056; .106 |  | -.010 (-.022; .002) | .006 | -.055; .114 |
| Physical activity | **-.400 (-.761; -.039)** | **.184** | **-.066; .030** |  | **-.402 (-.763; -.041)** | **.184** | **-.066; .029** |
| Lipids imbalance | **.739 (.203; 1.275)** | **.273** | **.081; .007** |  | **.704 (.153; 1.255)** | **.281** | **.078; .012** |
| Lipids imbalance X Age | --- | --- | --- |  | -.017 (-.077; .044) | .031 | -.017; .592 |
| F_(df1;df2)_; R^2^; R^2^_adjusted_; *p* | 23.271_(8; 934)_; .166; .159; <.001 | | |  | 20.701_(9; 933)_; .166; .158; <.001 | | |
| Age | .006 (-.037; .050) | .022 | .009; .775 |  | .004 (-.039; .047) | .022 | .006; .852 |
| Gender ^a^ | **-4.04 (-5.076; -3.004)** | **.528** | **-.317; <.001** |  | **-4.059 (-5.095; -3.024)** | **.528** | **-.318; <.001** |
| Formal education ^b^ | **-2.147 (-3.231; -1.062)** | **.553** | **-.123; <.001** |  | **-2.038 (-3.129; -.947)** | **.556** | **-.117; <.001** |
| Former smoker ^c^ | -.155 (-1.267; .957) | .567 | -.01; .784 |  | -.194 (-1.306; .918) | .567 | -.013; .732 |
| Smoker ^c^ | .398 (-1.269; 2.065) | .849 | .015; .639 |  | .395 (-1.27; 2.061) | .849 | .015; .642 |
| Alcohol consumption | -.009 (-.022; .003) | .006 | -.051; .149 |  | -.009 (-.022; .003) | .006 | -.050; .153 |
| Physical activity | **-.407 (-.77; -.044)** | **.185** | **-.067; .028** |  | **-.413 (-.775; -.050)** | **.185** | **-.068; .026** |
| Blood pressure | -.100 (-.672; .472) | .291 | -.011; .731 |  | -.075 (-.647; .497) | .292 | -.008; .797 |
| Blood pressure X Age | --- | --- | --- |  | -.053 (-.115; .010) | .032 | -.050; .098 |
| F_(df1;df2)_; R^2^; R^2^_adjusted_; *p* | 22.201_(8; 934)_; .160; .153; <.001 | | |  | 20.075_(9; 933)_; .162; .154; <.001 | | |

^a^ Gender, reference category: female. ^b^ Formal education measured in years, reference category: 4 years or less. ^c^ Smoking status, reference category: nonsmoker. ^d^ Alcohol consumption measured in gr/day, reference category: none. ^e^ Physical activity in number of times per week, reference category: none.

**Supplementary Table 3.** Linear regression the quadratic term of obesity in mood and moderation analysis of age.

| **GDS score** |  | | |  |  | | |
| --- | --- | --- | --- | --- | --- | --- | --- |
|  | **B (CI 95%)** | **SE** | **β; *p*** |  | **B (CI 95%)** | **SE** | **β; *p*** |
| Age | .002 (-.041; .045) | .022 | .003; .935 |  | .000 (-.043; .043) | .022 | .000; .993 |
| Gender ^a^ | **-3.922 (-4.961; -2.883)** | **.529** | **-.307; <.001** |  | **-3.992 (-5.029; -2.955)** | **.528** | **-.313; <.001** |
| Formal education ^b^ | **-2.063 (-3.145; -.981)** | **.551** | **-.119; <.011** |  | **-2.063 (-3.142; -.984)** | **.550** | **-.119; <.001** |
| Former smoker ^c^ | -.169 (-1.281; .943) | .567 | -.011; .766 |  | -.145 (-1.254; .964) | .565 | -.010; .798 |
| Smoker ^c^ | .321 (-1.342; 1.983) | .847 | .012; .705 |  | .509 (-1.153; 2.172) | .847 | .020; .548 |
| Alcohol consumption | -.010 (-.022; .002) | .006 | -.055; .110 |  | -.009 (-.021; .003) | .006 | -.051; .142 |
| Physical activity | **-.380 (-.742; -.018)** | **.184** | **-.063; .040** |  | **-.419 (-.781; -.057)** | **.184** | **-.069; .023** |
| Obesity | .134 (-.339; .607) | .241 | .018; .578 |  | .118 (-.356; .592) | .242 | .016; .626 |
| Obesity^2^ | **.434 (.099; .769)** | **.171** | **.081; .011** |  | **.468 (.115; .82)** | **.180** | **.087; .009** |
| Obesity X Age | --- | --- | --- |  | **-.077 (-.131; -.023)** | **.027** | **-.090; .005** |
| Obesity^2^ X Age | --- | --- | --- |  | .021 (-.021; .064) | .022 | .033; .331 |
| F_(df1;df2)_; R^2^; R^2^_adjusted_; *p* | 22.884_(8; 934)_; .164; .157; <.001 | | |  | 17.872_(11; 931)_; .174; .165; <.001 | | |
